# Chronic Diarrhea and Alcoholism: Unravelling the Connection to Pellagra

**DOI:** 10.7759/cureus.82088

**Published:** 2025-04-11

**Authors:** Jayashree Ravikumar, Divya Ravikumar

**Affiliations:** 1 Internal Medicine, Sri Varadhar Consultation Clinic, Chennai, IND; 2 Obstetrics and Gynecology, The Hive Fertility and Women's Centre, Chennai, IND

**Keywords:** dermatitis, diarrhea, niacin deficiency, pellagra, vitamin b3 deficiency

## Abstract

Pellagra is a deadly systemic disease caused by niacin deficiency or a disorder of its metabolism. It typically presents as a classic triad of diarrhea, dermatitis, and dementia. It is increasingly seen among high-risk populations such as those with poor socioeconomic status, immunocompromised states, chronic alcoholism, malabsorption disorders, and populations heavily reliant on a corn-based diet. We report a case of a 39-year-old man presenting with watery diarrhea and hyperpigmentation over the sun-exposed areas of the face and arms, with a history of alcohol use disorder. Differential diagnoses were excluded. Niacinamide supplements and lifestyle modifications were recommended, resulting in the resolution of the symptoms. This case report emphasizes the need for a high index of suspicion when diagnosing pellagra in vulnerable populations to ensure prompt treatment and recovery.

## Introduction

Pellagra is a niacin deficiency disorder characterized by a triad of dermatitis, diarrhea, and dementia [1]. Niacin is an important cofactor in several oxidation and reduction reactions involved in cellular metabolism and respiration [2]. The Recommended Dietary Allowance (RDA) for adults is 16 mg/day of niacin equivalents (NEs) for men and 14 mg/day of NEs for women [3]. Although it has been eradicated in developed countries, it is still prevalent among high-risk populations such as alcoholics, the malnourished, individuals on immunosuppressive drugs, and those with infections or drug abuse [4]. Chronic alcoholism has become the primary cause of pellagra in this era [5]. Chronic diarrhea is defined as diarrhea that persists for more than four weeks, with differential diagnoses including irritable bowel syndrome, inflammatory bowel disease, endocrine disorders, and food allergies or sensitivities [6]. Although pellagra is within the differential diagnosis, it is often overlooked, leading to delays in diagnosis and treatment. This case highlights the importance of evaluating vulnerable populations for pellagra.

## Case presentation

**Figure 1:**
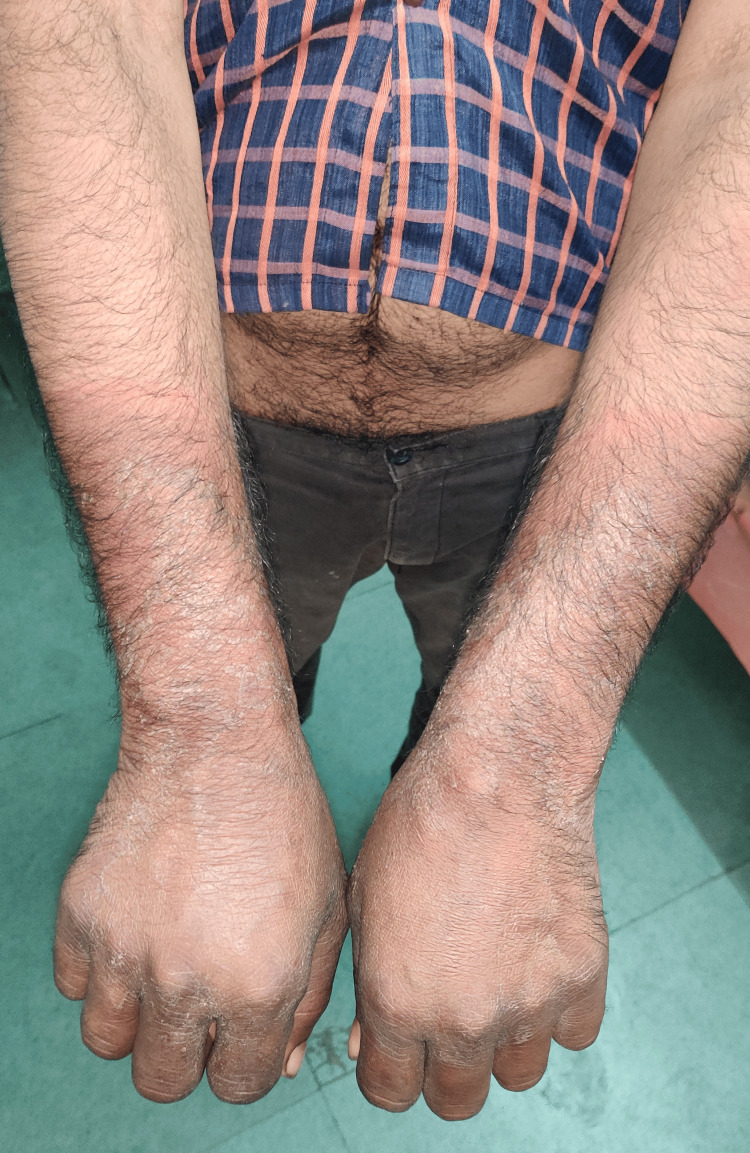
Dermatitis secondary to pellagra, suggested by dry, dark, and scaly skin over the arms of the patient

**Figure 2:**
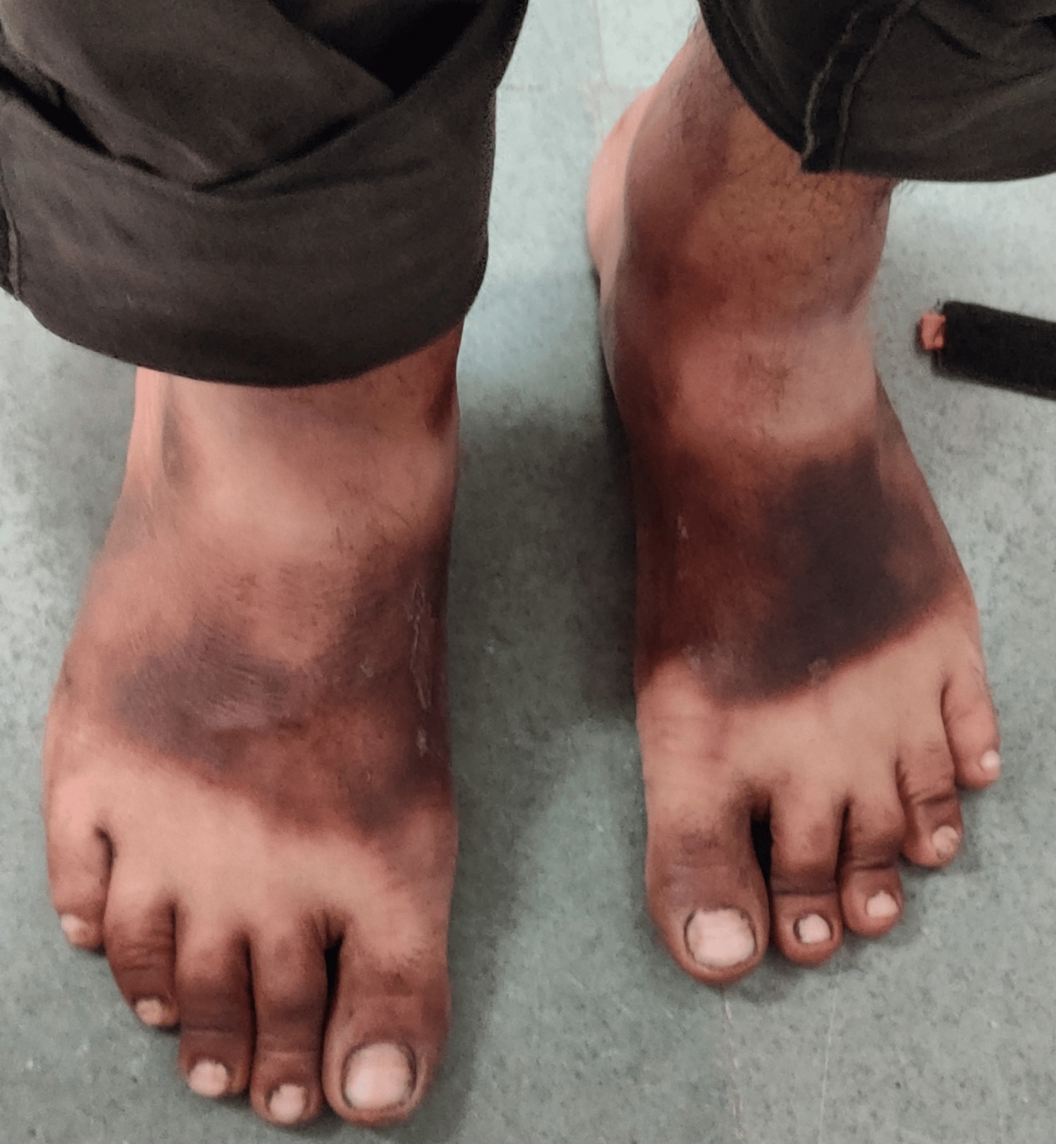
Dermatitis secondary to pellagra, suggested by dry, dark, and scaly skin over the feet of the patient

**Figure 3:**
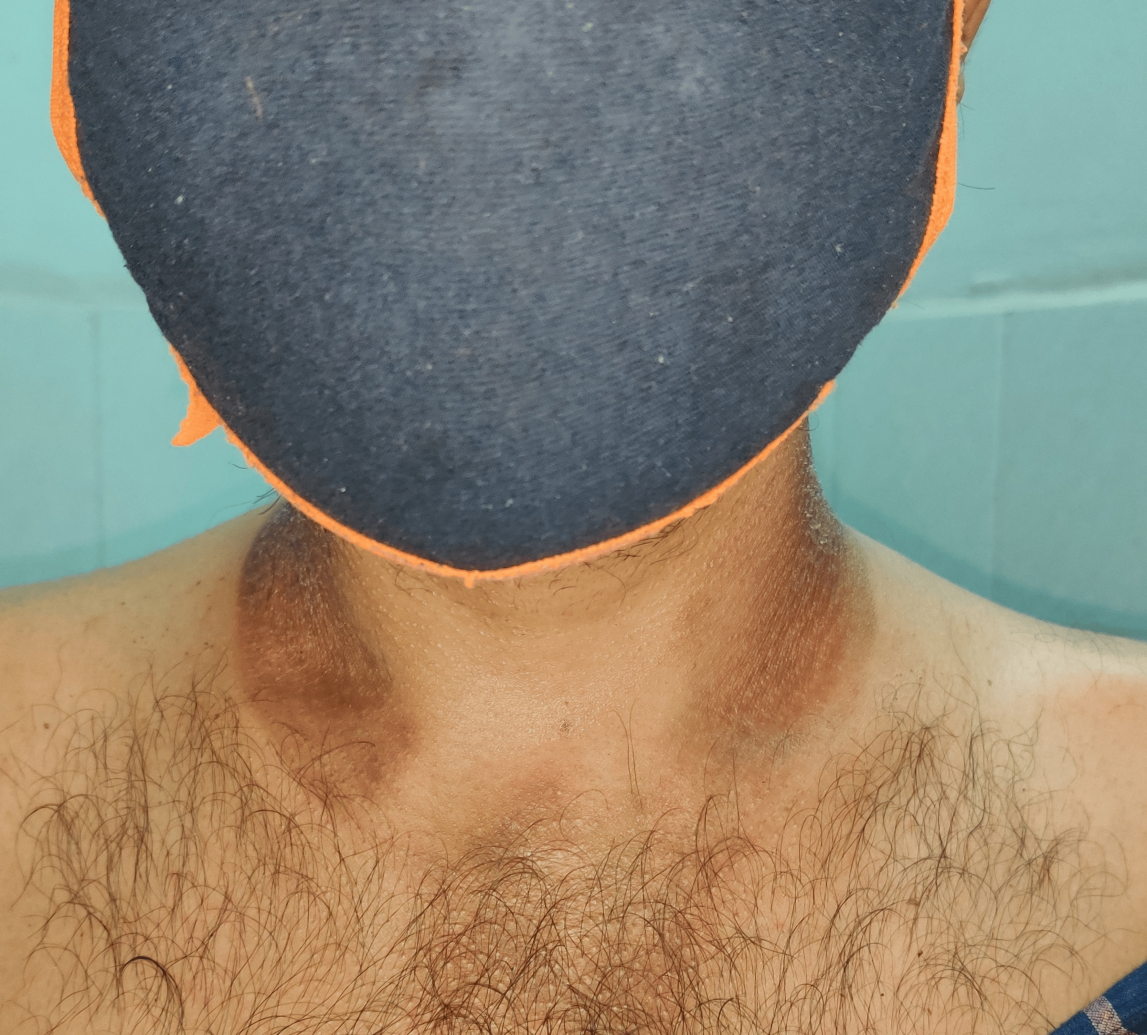
Casal collar hyperpigmentation of the neck

A 39-year-old South Asian man presented with chronic diarrhea for seven months. He had eight watery stools daily over this period, resulting in a weight loss of 18 kg. He also complained of malaise and pruritus. He had no fever, cough, night sweats, or neurological symptoms. He had a 20-pack-year history of smoking and had an alcohol use disorder for 10 years, with an estimated consumption of 40-50 g per day. On admission, his vital signs were as follows: temperature of 98.4°F, heart rate of 102 bpm, respiratory rate of 15 bpm, and blood pressure of 106/58 mm Hg. He weighed 48 kg with a BMI of 17.2 kg/m². He appeared conscious, oriented, cachectic, and dehydrated. On examination, a hyperpigmented, dry, thickened, desquamating rash was observed on sun-exposed areas of the forehead, arms, and feet (Figures 1, 2). Casal collar hyperpigmentation was noted on the neck (Figure 3). Cardiovascular and respiratory examinations did not reveal any significant findings. Abdominal examination revealed ascites; the abdomen was soft and scaphoid, with the liver not palpable.

Laboratory investigations revealed microcytic hypochromic anemia (Table 1). Common causes such as chronic infections (tuberculosis, hepatitis, HIV) and malignancies were ruled out through targeted testing. The patient presented with a photosensitive rash over the neck resembling Casal’s necklace, chronic diarrhea, and a history of chronic alcohol abuse, all consistent with a clinical diagnosis of pellagra. This diagnosis was made based on characteristic dermatological and gastrointestinal findings in conjunction with the patient’s risk factors. Nutritional assessment revealed a severely deficient diet, with daily niacin intake estimated at less than 5 mg/day, well below the RDA of 16 mg/day for adult males. Caloric intake was low, with a predominance of maize-based foods lacking adequate protein or B vitamins. The patient was not consuming fortified foods or supplements.

**Table 1:** Laboratory Result of complete hemogram in the patient with pellagra at the time of diagnosis

Laboratory Test (Unit)	Results	Reference value
Erythrocyte (red blood cells) count (million/µL)	4.0	3.80-4.80
Hemoglobin (g/dL)	8.8	13-17
Packed cell volume (%)	30	36.0-46.0
Mean corpuscular volume (fL)	78	83.0-101.0
Mean corpuscular hemoglobin (pg)	28	27.0-32.0
Mean corpuscular hemoglobin concentration (g/dL)	32	31-34.5
Red cell distribution width (%)	11	11.6-14.0
White blood cells (cells/µL)	8.8	4000-11000
Platelets (10^9^/L)	200	150-450
Peripheral smear	Microcytic hypochromic anemia	

Treatment included nicotinamide 100 mg orally every six hours for seven days. A nutritionally balanced hospital diet includes approximately 2,200 kcal/day; niacin (including nicotinamide and dietary sources): ~80-100 mg/day; adequate intake of vitamin C, B-complex vitamins, and proteins; hydration: 2-2.5 L/day orally, and topical sunscreen for photoprotection. In parallel, the patient was enrolled in an alcohol rehabilitation program, receiving behavioral therapy and social support. Financial aid was arranged to ensure food security and access to treatment. A dedicated counselor was assigned for continuous monitoring and emergency support. After three weeks of treatment, the patient showed marked clinical improvement. Rash and diarrhea resolved gradually. Hemoglobin levels improved gradually (Table 2). Appetite and overall nutritional status improved. He reported abstinence from alcohol, confirmed by counseling reports.

**Table 2:** : Laboratory result of complete hemogram of the patient after treatment

Laboratory tests (units)	Results	Reference values
Erythrocyte (red blood cells) count (million/µL)	4.4	3.80-4.80
Hemoglobin (g/dL)	13.8	13-17
Packed cell volume (%)	38	36.0-46.0
Mean corpuscular volume (fL)	85	83.0-101.0
Mean corpuscular hemoglobin (pg)	29	27.0-32.0
Mean corpuscular hemoglobin concentration (g/dL)	33	31-34.5
Red cell distribution width (%)	13.1	11.6-14.0
White blood cells (cells/µL)	9.7	4000-11000
Platelets (10^9^/L)	260	150-450

## Discussion

Globally, pellagra was prevalent in the early 20th century in Europe and the USA, linked to corn-based diets lacking proper niacin or tryptophan [7].

In India, a retrospective analysis of 335 patients (1992-2012) reported pellagra mostly in males aged 30-40, with chronic energy deficiency noted in 63.8% of cases [8]. Historical data from Calcutta in 1935 documented pellagra in poor Hindu widows, emphasizing the role of dietary and social deprivation [9].

In South Africa, pellagra remained a public health concern into the late 20th century, particularly among maize-dependent populations. A 2019 scoping review identified 15 major pellagra outbreaks from 1897 to 2019 [10].

Nicotinamide is a natural derivative of niacin. Niacin, a component of coenzymes, is used in oxidation and reduction reactions essential for cellular metabolism. It is involved in the cellular processing of proteins, carbohydrates, and fats [4]. Tissues with high regeneration rates and high energy consumption, such as the skin, intestines, and brain, can be particularly affected by niacin deficiency [11].

The disease is classically defined by the four “D’s”: dermatitis, diarrhea, dementia, and death. First, dermatitis occurs in sun-exposed areas with sharp borders. It starts as a red rash and then progresses to hyperpigmented, itchy, and scaly patches. On the neck, lesions appear in a pattern resembling a necklace, earning the name “Casal’s necklace” [12].

Second, diarrhea is not a common symptom of pellagra but can occur due to inflammation of the gastrointestinal (GI) mucosa. Depending on the location of the inflammation along the GI tract, symptoms can vary. It can also present as glossitis, nausea, vomiting, abdominal pain, and malabsorption [1].

Third, dementia is an advanced symptom after which death occurs. Neuropsychiatric changes such as headache, irritability, poor concentration, anxiety, delusions, hallucinations, photophobia, tremor, ataxia, spastic paresis, fatigue, and depression can progress to encephalopathy, characterized by confusion, memory loss, delirium, stupor, and coma [4]. While often diagnosed clinically, atypical presentations may be confirmed by measuring levels of N-methylnicotinamide and 2-pyridone [13].

Treatment involves administering niacin and incorporating a niacin-rich diet that includes yeast, eggs, bran, peanuts, meat, poultry, fish, legumes, and whole-grain cereals. The resolution of symptoms will be gradual. Supportive management, such as adequate hydration (up to two to three liters of fluids) and avoiding sun exposure with sunscreen, is advised [14]. Pellagra due to alcohol use disorder requires additional treatment, which may involve addiction therapy or psychotherapy, depending on specialist consultation [15].

## Conclusions

Pellagra, though eradicated in developed countries, is reemerging in vulnerable populations. Chronic diarrhea and alcoholism might initially appear unrelated but can together contribute to pellagra. Understanding this connection is crucial for timely diagnosis and prompt treatment. A holistic approach includes correcting the deficiency through dietary modifications and niacin supplementation, managing contributing factors like GI disorders, providing support for coexisting conditions such as alcohol use disorder, and offering symptomatic care to help resolve the condition promptly.

## References

[1] Cao S, Wang X, Cestodio K (2020). Pellagra, an almost-forgotten differential diagnosis of chronic diarrhea: more prevalent than we think. Nutr Clin Pract.

[2] Kirkland JB, Meyer-Ficca ML (2018). Niacin. Advances in Food and Nutrition Research.

[3] Institute of Medicine (1998). Niacin, Vitamin B6, Folate, Vitamin B12, Pantothenic Acid, Biotin, and Choline. Dietary Reference Intakes for Thiamin, Riboflavin, Niacin, Vitamin B6, Folate, Vitamin B12, Pantothenic Acid, Biotin, and Choline.

[4] Hegyi J, Schwartz RA, Hegyi V (2004). Pellagra: dermatitis, dementia, and diarrhea. Int J Dermatol.

[5] Navari Y, Bagheri A, Foreback J (2023). A rare case of pellagra in a chronic alcoholic. Cureus.

[6] Mills K, Akintayo O, Egbosiuba L (2020). Chronic diarrhea in a drinker: a breakthrough case of pellagra in the US South. J Investig Med High Impact Case Rep.

[7] Rajakumar K (2000). Pellagra in the United States: a historical perspective. South Med J.

[8] Sharma L, Tiwari S, Kalhan A (2019). Pellagra: a forgotten entity. Curr Drug Discov Res.

[9] Sen Gupta PC, Rai Chaudhuri MN, Chaudhuri RN (1939). Notes on cases of pellagra encountered in Calcutta. Ind Med Gaz.

[10] Viljoen M, Bipath P, Tosh C (2021). Pellagra in South Africa from 1897 to 2019: a scoping review. Public Health Nutr.

[11] Usman AB, Emmanuel P, Manchan DB, Chinyere A, Onimisi OE, Yakubu M, Hirayama K (2019). Pellagra, a re-emerging disease: a case report of a girl from a community ravaged by insurgency. Pan Afr Med J.

[12] Pinheiro H, Matos Bela M, Leal AF, Nogueira L, Mesquita M (2021). Hidden hunger: a pellagra case report. Cureus.

[13] Delgado-Sanchez L, Godkar D, Niranjan S (2008). Pellagra: rekindling of an old flame. Am J Ther.

[14] Segula D, Banda P, Mulambia C, Kumwenda J (2012). Case report-a forgotten dermatological disease. Malawi Med J.

[15] Hołubiec P, Leończyk M, Staszewski F, Łazarczyk A, Jaworek AK, Wojas-Pelc A (2021). Pathophysiology and clinical management of pellagra-a review. Folia Med Cracov.

